# Supplementary material for: Metabolic activity and survival strategies of thermophilic microbiomes during hyperthermophilic composting
Source: mSystems. 2025 Oct 23;10(11):e00956-25. doi: 10.1128/msystems.00956-25 (PMC12625702; doi:10.1128/msystems.00956-25)
Supplement: Supplemental material — Supplemental text and figures. [file msystems.00956-25-s0001.docx]

**Metabolic activity and survival strategies of thermophilic microbiomes during hyperthermophilic composting**

Chen Liu^1#^, Yuqi He^1#^, Hongbo Zhang ^1^, Dong Zhang^1^, Chaofan Ai^1^, Xiang Tang^1^, Qiu-e Yang^1^, Zhen Yu^2^, Shiyong Tan^3^, Ville-Petri Friman^4^, Hanpeng Liao^1*^, Shungui Zhou^1^

**Affiliations**

^1^ Fujian Provincial Key Laboratory of Soil Environmental Health and Regulation, College of Resources and Environment, Fujian Agriculture and Forestry University, Fuzhou 350002, China.

^2^ Research Center for Eco-Environmental Engineering, Dongguan University of Technology, Dongguan 523808, China

^3^ Yuelushan Laboratory, Changsha, 410128, China

^4^ Department of Microbiology, University of Helsinki, Helsinki, 00014, Finland

**^#^** Chen Liu and Yuqi He contributed equally to this work.

**^*^Corresponding authors**

Correspondence to Hanpeng Liao ([liaohp@fafu.edu.cn](mailto:liaohp@fafu.edu.cn))

**Supplementary methods**

**Text S1. Quantifying and analyzing bacterial communities based on 16S rRNA amplicon sequencing**

The 25 µL PCR mixture containing 10 µL of 2.5 5×Prime HotMaster mix (5Prime, Gaithersburg, MD), 13 µL of ddH_2_O (Mo Bio Laboratories, Carlsbad, CA, USA), 0.5 µL of each primer (10 µM) and 1 µL of isolated DNA was used as the template. Primers were designed for Illumina sequencing with adapters, primer pads, and 2-bp linker sequences, and the reverse primer contained a 12-bp barcode sequence unique to each sample. The thermal cycling protocol was 94°C for 5 min, 35 cycles of 94°C for 45 s, 50°C for 45 s, and 72°C for 90 s, followed by a final extension period at 72°C for 10 min. All samples were amplified in triplicate on thermal cyclers (Bio-Rad, Hercules, CA, USA), pooled together, and visualized on agarose gels. PCR products were purified using the UltraClean PCR cleanup kit (Mo Bio Laboratories Cleveland, OH, USA). The universal primers 515F and 907R targeting the V4-V5 region of the 16S rRNA gene was used. The raw 16S rRNA gene sequences were processed using QIIME 2 (version 2019.7)^3^. Raw reads were quality filtered (i.e. filtered, dereplicated, denoised, merged, and assessed for chimaeras) to produce amplicon sequence variants (ASV) using the Dada2 pipeline via QIIME2^4^. The DADA2 generated feature table was filtered to remove ASVs at a frequency less than two, and remaining ASV were classified using the QIIME2 naive Bayes classifier trained on 99% operational taxonomic units from the SILVA rRNA database (v 132)^5^. The microbial diversity and richness were estimated using alpha diversity (Shannon and observed ASV) and beta diversity (weighted UniFrac distance) using the q2-diversity pipeline within QIIME2.


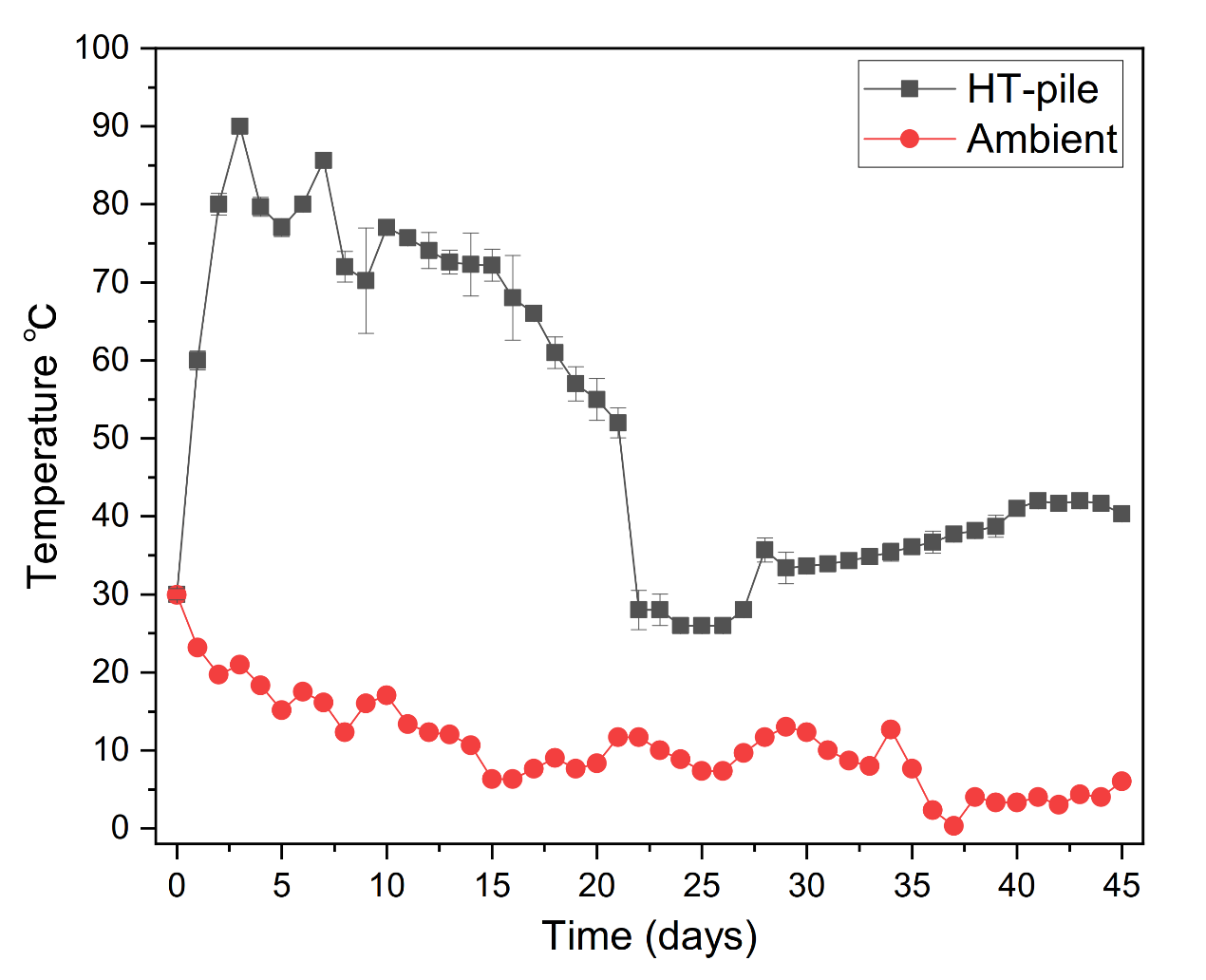


**Figure S1.** The temperature profile of hyperthermophilic composting in the full-scale experiments. The sewage sludge of the hyperthermophilic composting plant covered 45, 000 m^2^ and at GeoGreen Innotech Co., Ltd., in Beijing, China. This factory has the ability to deal with 600 tons of dewatered sewage sludge (80% of moisture content) per day and the average maximum fermentation temperatures of composting piles are around 90°C.


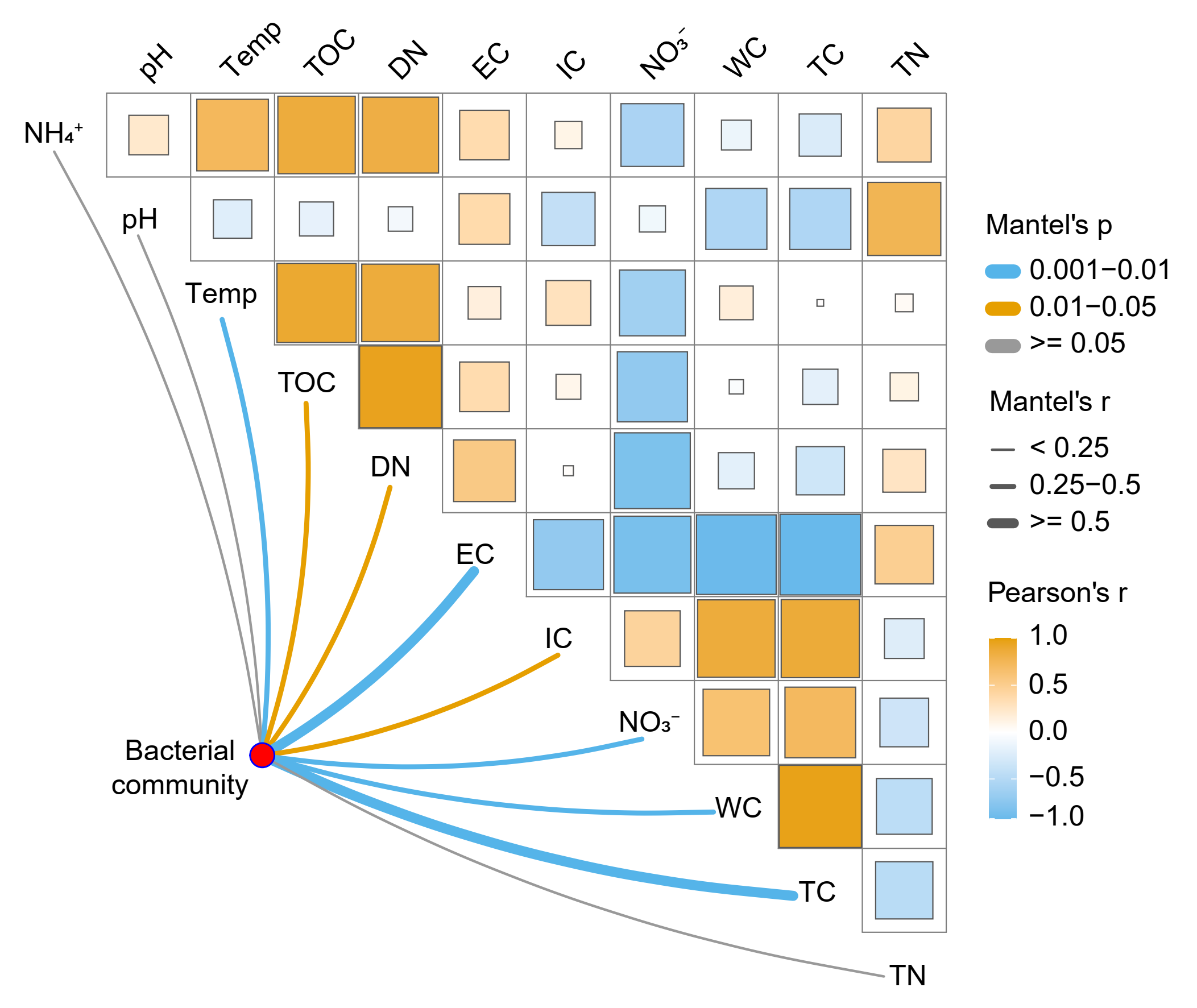


**Figure S2.** Correlations between physicochemical properties and bacterial community composition based on 16S rRNA gene amplicon sequencing. For the Mantel test, bacterial community composition was represented by a Bray–Curtis dissimilarity matrix calculated from genus-level relative abundances (>0.1%), and physicochemical properties were represented by Euclidean distance matrices. Edge width represents the absolute value of the Mantel correlation coefficient, and edge color indicates statistical significance. gradients denoting Pearson’s correlation coefficients. Abbreviations: ammonium (NH₄⁺); Temperature (Temp) The upper triangular matrix shows pairwise correlations among physicochemical properties, with color; total organic carbon (TOC); dissolved total nitrogen (DN); electrical conductivity (EC); inorganic carbon (IC); nitrate (NO₃⁻); water content (WC); total carbon (TC), total nitrogen (TN).


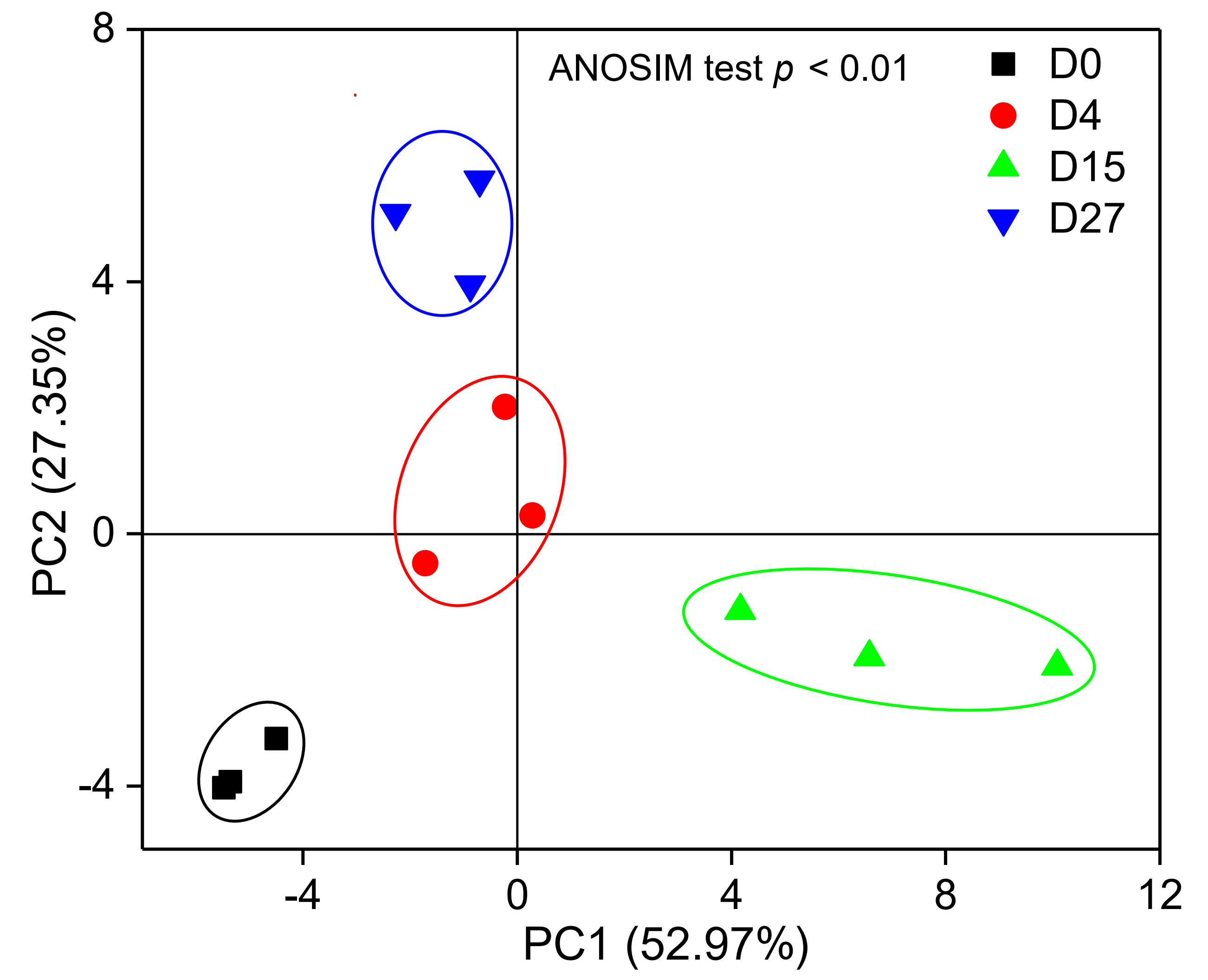


**Figure S3.** Changes in the microbial functions based on KEGG Orthologs (KOs) at different phases during the hyperthermophilic composting (HTC) based on PCoA Analysis. D0, D4, D15, and D27 refer to samples collected on days 0, 4, 15, and 27 of the composting process.


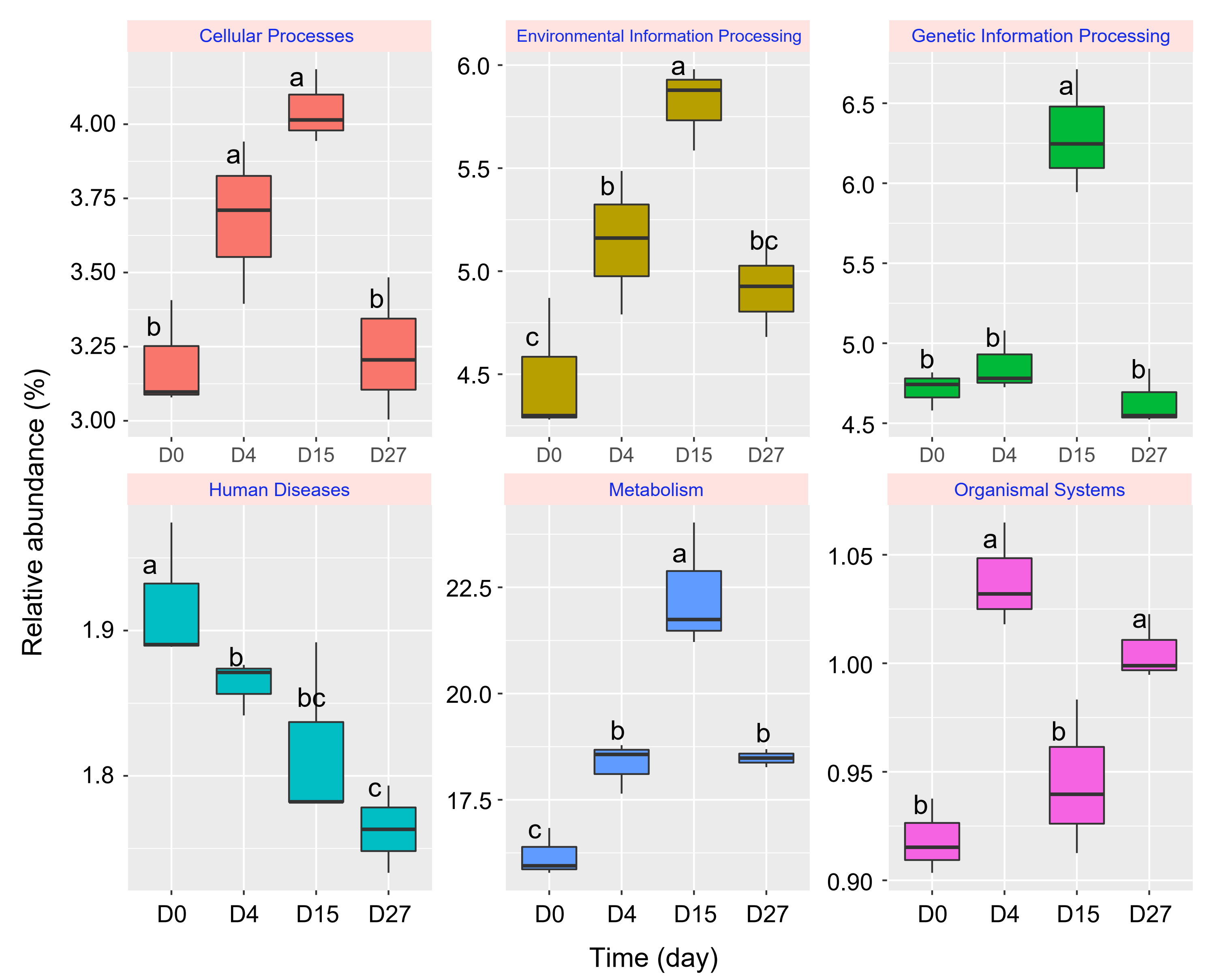


**Figure S4.** Changes in the microbial functional genes based on KEGG Orthologs (KOs at level 1) at different phases during the hyperthermophilic composting (HTC). In all panels, data are presented as mean ± SD (n =3) of three biologically independent replicates, with different lowercase letters between treatments indicating significant pairwise differences at levels: p = 0.05 (n.s: no significant difference). D0, D4, D15, and D27 refer to samples collected on days 0, 4, 15, and 27 of the composting process.


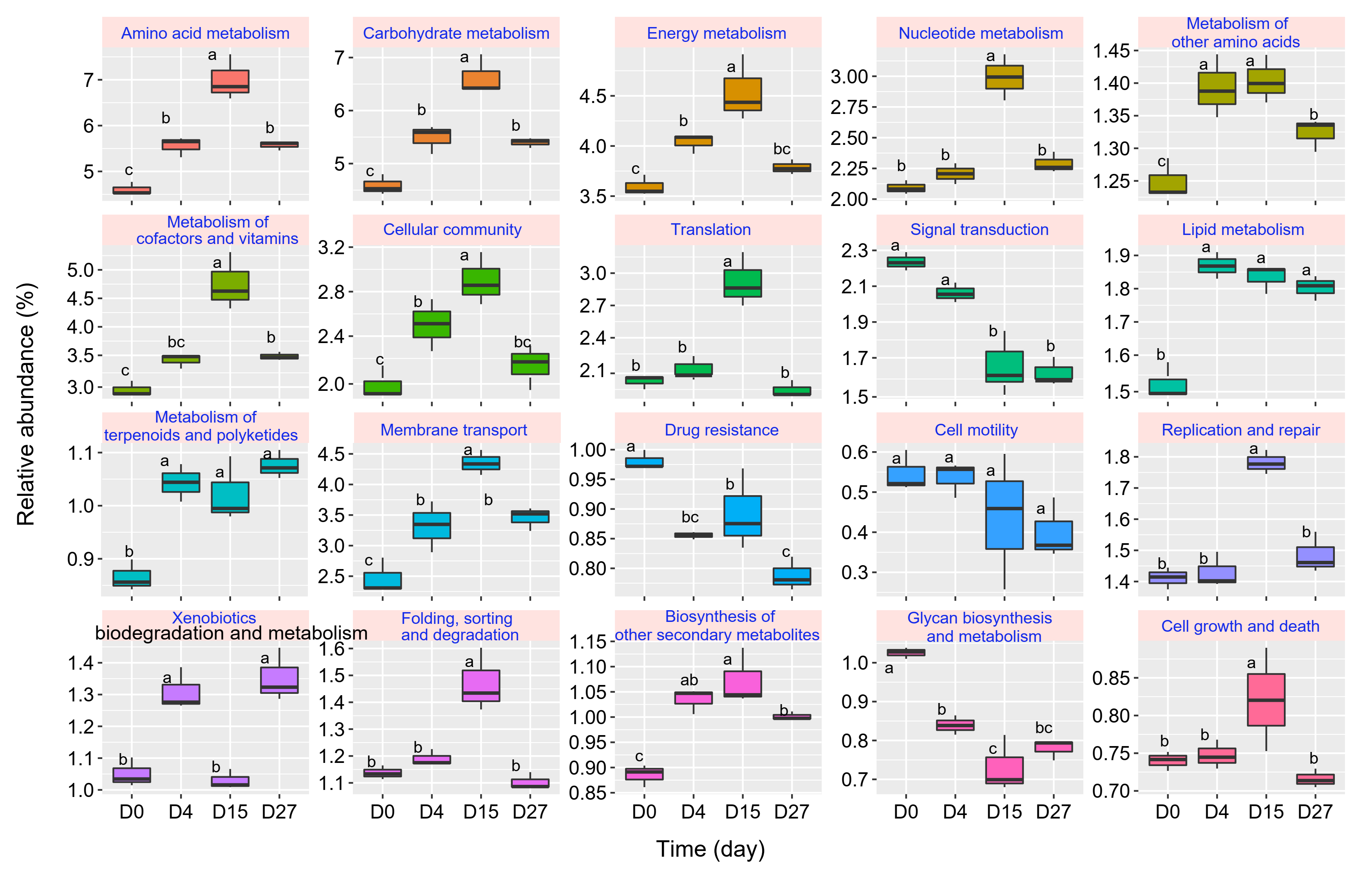


**Figure S5.** Changes in the microbial functional genes based on KEGG Orthologs (KOs at level 2) at different phases during the hyperthermophilic composting (HTC). In all panels, data are presented as mean ± SD (n = 3) of three biologically independent replicates, with different lowercase letters between treatments indicating significant pairwise differences at levels: p = 0.05 (n.s: no significant difference). D0, D4, D15, and D27 refer to samples collected on days 0, 4, 15, and 27 of the composting process.


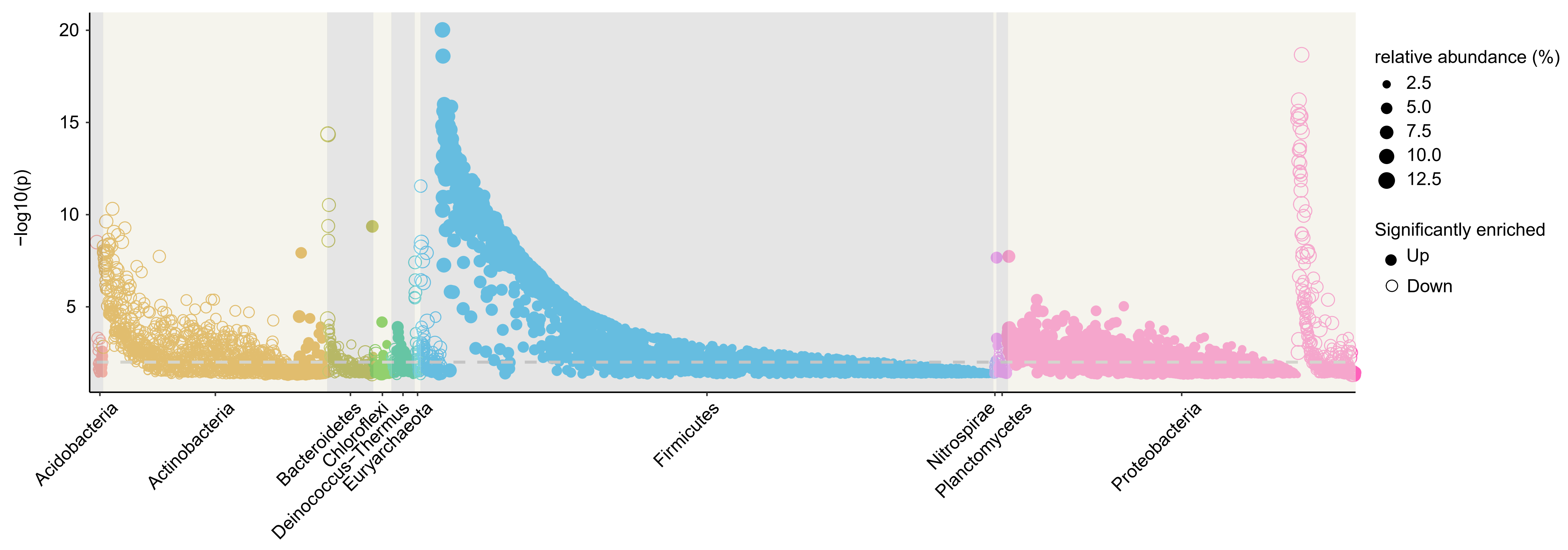


**Figure S6.** Changes in the functional genes at the thermophilic phase (D15) compared to the initial phase (D0) in the dominant bacterial taxa at the phylum level. The circle size represents the relative abundance of functional genes. Hollow circles indicate the significant enrichment of functional gene abundance at the thermophilic phase, while solid circles indicate significant decrease in functional gene abundance.


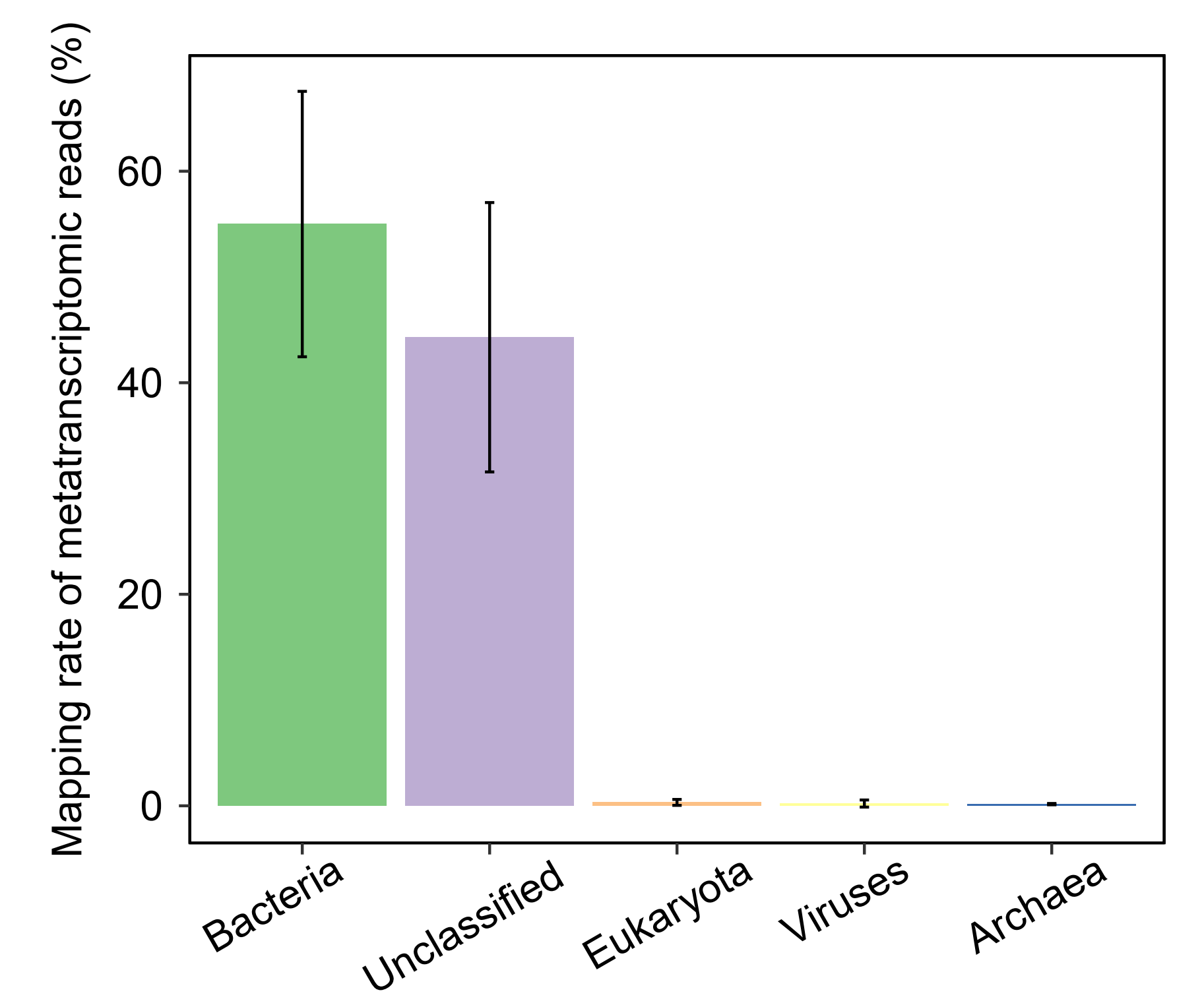


**Figure S7.** Composition of different active microorganisms revealed by metatranscriptomic sequencing during the HTC.


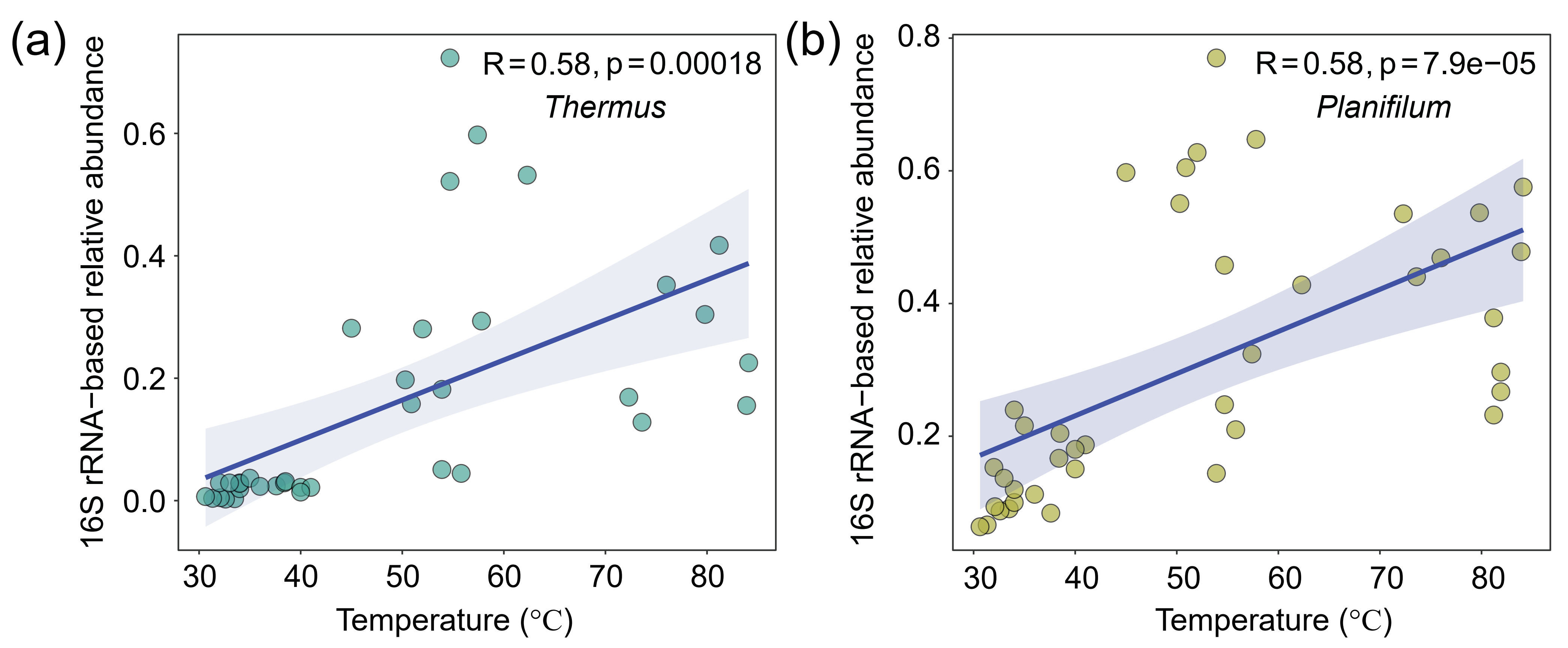
**Figure S8.** Correlation between composting temperature and the 16S rRNA-based relative abundance of thermophilic bacteria. (a) *Thermus* and (b) *Planifilum* show increasing relative abundance with rising composting temperature. Shaded areas indicate 95% confidence intervals of the linear regression fit.


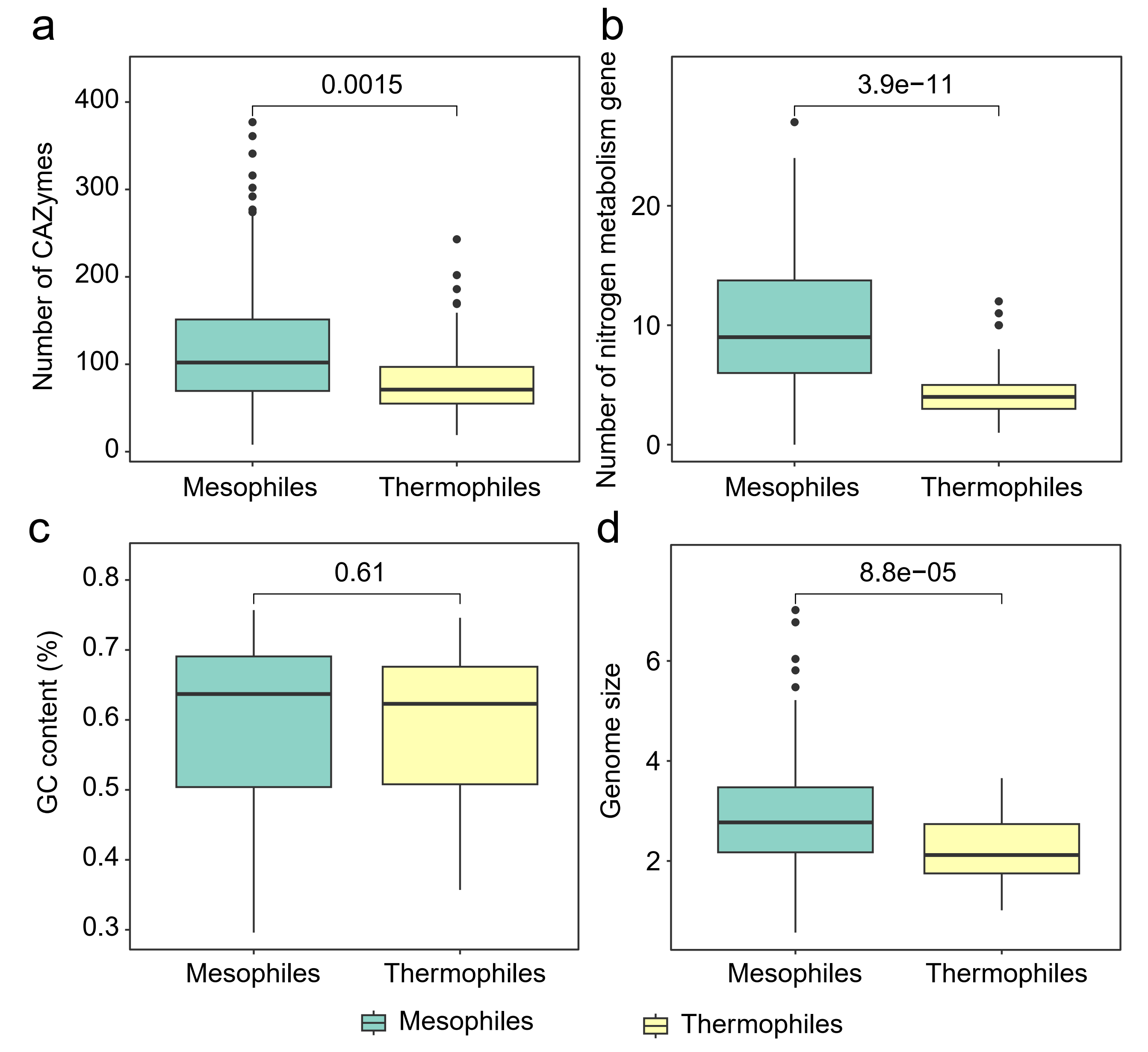


**Figure S9.** Differences in number of carbohydrate-active enzymes (CAZymes, a) and nitrogen metabolism-related genes (identified from the KEGG database, b), GC content (c), and genome size (d) in the mesophilic and thermophilic MAGs.


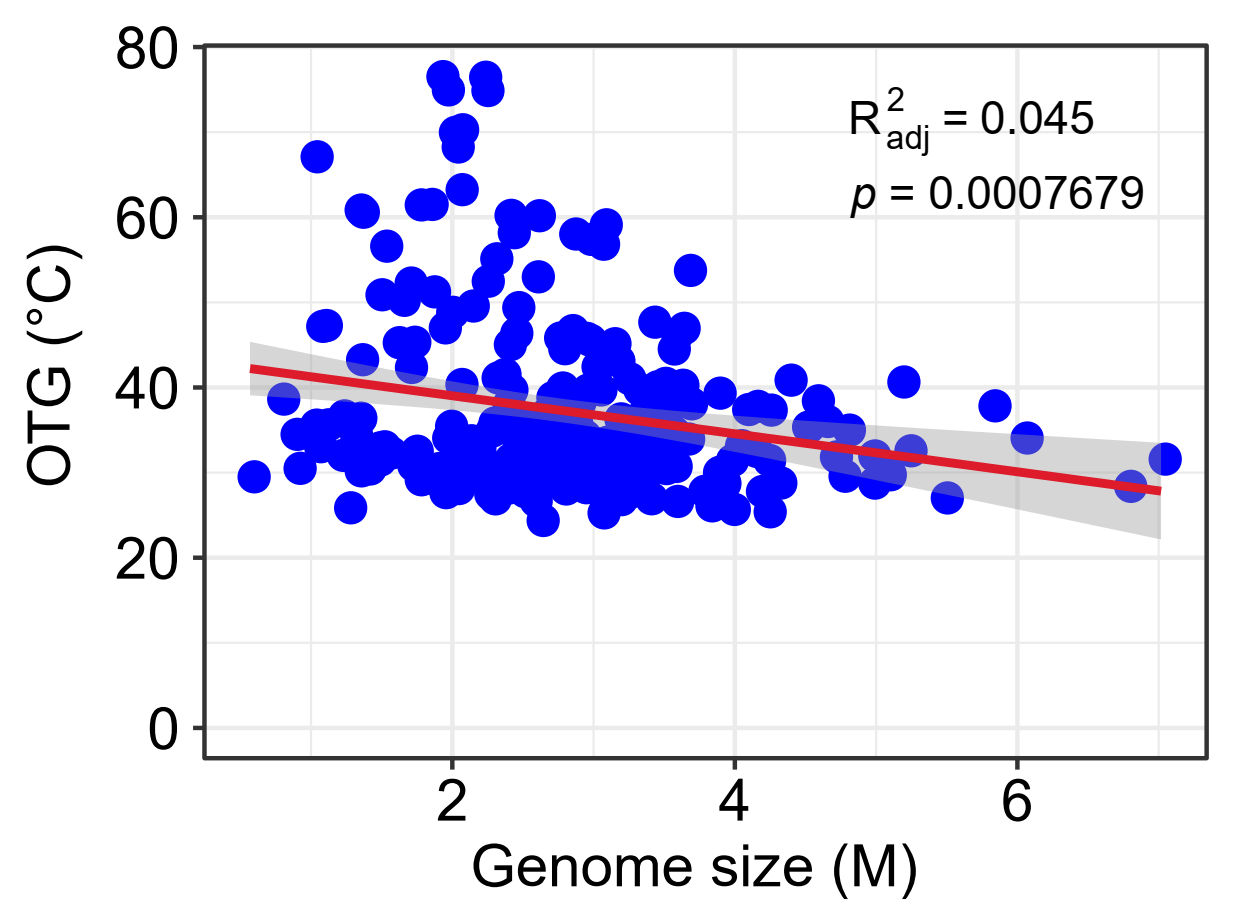


**Figure** **S10.** Genome size is significantly correlated with optimal growth temperature (OGT) in all MAGs.


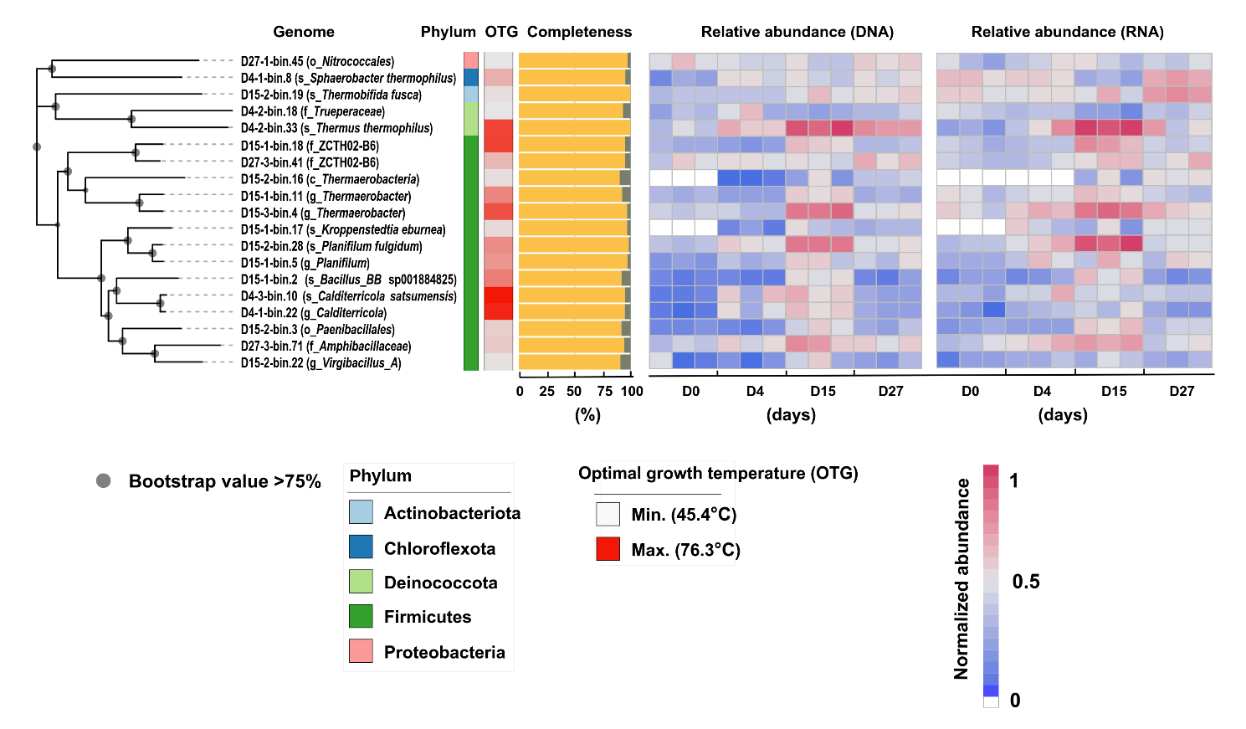


**Figure S11.** Refined thermophilic MAGs of phylogeny, assembly statistics, the optimal growth temperature (OGT), and abundance at different phases based on metagenomic (DNA level) and metatranscriptomic (RNA level). Tree scale represents estimated site divergence and green dots indicate branching calculated with bootstrapped conﬁdence >75%. Barplot shows the completeness (yellow) and contamination (grey) determined by CheckM. Relative abundance of MAGs calculated by alignment of quality-filtered reads against refined genomes using BBMap based on metagenomic and metatranscriptomic reads. Abundance normalized using feature scaling between 0 and 1 for each genome. D0, D4, D15, and D27 refer to samples collected on days 0, 4, 15, and 27 of the composting process.
